# Dual antiplatelet therapy does not affect the incidence of low-dose aspirin-induced small intestinal mucosal injury in patients after percutaneous coronary intervention for coronary stenosis: a multicenter cross-sectional study

**DOI:** 10.3164/jcbn.18-16

**Published:** 2018-05-25

**Authors:** Azusa Hara, Kazuhiro Ota, Toshihisa Takeuchi, Yuichi Kojima, Yuki Hirata, Haruhiko Ozaki, Shinpei Kawaguchi, Yoshiaki Takahashi, Satoshi Harada, Taisuke Sakanaka, Takeshi Ogura, Sadaharu Nouda, Kazuki Kakimoto, Ken Kawakami, Akira Asai, Shinya Fukunishi, Makoto Sanomura, Kazunari Tominaga, Kazuhide Higuchi

**Affiliations:** 1 Second Department of Internal Medicine, Osaka Medical College, 2-7 Daigaku-machi, Takatsuki, Osaka 569-8686, Japan; 2 Department of Gastroenterology, Hokusetsu General Hospital, 6-24 Kitayanagawa-cho, Takatsuki, Osaka 569-8585, Japan; 3 Premier Developmental Research of Medicine, Osaka Medical College, 2-7 Daigaku-machi, Takatsuki, Osaka 569-8686, Japan

**Keywords:** aspirin, clopidogrel, proton pump inhibitor, capsule endoscopy, small intestinal lesion

## Abstract

Although low-dose aspirin (LDA) is known to induce small intestinal mucosal injury, the effect of dual antiplatelet therapy (DAPT; LDA + clopidogrel) on small intestinal mucosa in patients after percutaneous coronary intervention (PCI) for coronary stenosis is unknown. Fifty-one patients with a history of PCI and LDA use were enrolled, and 45 eligible patients were analyzed. Patients were grouped based on DAPT (DAPT: *n* = 10 and non-DAPT: *n* = 35) and proton pump inhibitor (PPI) use (PPI user: *n* = 22 and PPI-free patients: *n* = 23) to compare small intestinal endoscopic findings. The relationship between LDA-use period and small intestinal endoscopic findings was also examined. Multivariate analysis was performed to identify risk factors for LDA-induced mucosal injury using age, sex, DAPT, PPI, gastric mucoprotective drug, and LDA-use period. The rate of small intestinal mucosal injury incidence did not significantly differ between DAPT and non-DAPT patients (50% vs 51.1%, respectively; *p* = 0.94), or PPI users and PPI-free patients (50% vs 52.2%, respectively; *p* = 0.88). Additionally, LDA-use period of ≤24 months (*n* = 15) yielded a significantly higher rate of small intestinal mucosal injury incidence than LDA-use period >24 months (*n* = 30) (80% vs 36.7%, respectively; *p* = 0.006). Multivariate analysis revealed that a LDA-use period of ≤24 months was a significant risk factor for small intestinal mucosal injury (odds ratio: 19.5, 95% confidence interval: 2.48–154.00, *p* = 0.005). Following PCI for coronary stenosis, neither DAPT nor PPI affected LDA-induced small intestinal mucosal injury. Moreover, patients who used LDA within the last 24 months were at a greater risk of small intestinal mucosal injury.

## Introduction

The development of percutaneous coronary intervention (PCI) widened the indications for coronary stenosis treatment.^(1)^ In addition, the introduction of the drug-eluting stent has decreased restenosis of the coronary arteries.^(2)^ However, because drug-eluting stent placement harbors a risk of thrombosis within the stent, patients must undergo dual antiplatelet therapy (DAPT), typically consisting of low-dose aspirin (LDA) and clopidogrel, for a fixed period postoperatively. LDA is important to prevent primary and secondary thrombosis in patients with arteriosclerosis.^(3,4)^ Unfortunately, LDA-induced small intestinal mucosal injury is often a cause of anemia in LDA users.^(5–11)^

LDA is widely known to exert antiplatelet effects by inhibiting cyclooxygenase-1 activity, and suppressing the production of prostaglandins. However, this reduction of prostaglandins causes gastrointestinal mucosal injury.^(12)^ Watanabe *et al.*^(5)^ reported that over 90% of LDA users with upper gastrointestinal mucosal injury had LDA-induced small intestinal mucosal injury. Although the mechanisms by which clopidogrel use leads to worsening LDA-induced small intestinal mucosal injuries are unknown in humans, a previous study in animals revealed that platelet adenosine diphosphate-receptor antagonists, such as clopidogrel, impair the healing of gastric ulcers by suppressing the release of platelet-derived growth factors.^(13)^ Fork *et al.*^(14)^ reported that clopidogrel was not a risk factor of upper gastrointestinal mucosal injury in healthy volunteers, though in a retrospective study by Shiotani *et al.*,^(15)^ DAPT exacerbated small intestinal mucosal injury.

Generally, it has been recommended that LDA users concurrently use a proton pump inhibitor (PPI) for the prevention of upper gastrointestinal mucosal injury;^(16,17)^ however, PPIs cannot prevent small intestinal mucosal injury because gastric acid is not present in the small intestine.

If patients with poor cardiac function or severe coronary artery stenosis suffer severe anemia, their cardiac function could deteriorate and, in some cases, threaten their lives. It was previously reported that gastrointestinal bleeding in patients after PCI tended to be fatal.^(18)^ Therefore, it is important to understand the risk factors of LDA-induced small intestinal mucosal injury, and to intervene therapeutically as a general management strategy for patients with ischemic heart disease. Although there are several studies on LDA-induced small intestinal mucosal injury in healthy volunteers, data in actual patients are limited.^(5–11)^ While Watanabe *et al.*^(5)^ reported a frequency of small intestinal mucosal injury in patients that exceeded 90%, studies in healthy volunteers have observed rates ranging from 0–64%.^(8,10,19,20)^ Thus, patients with severe arteriosclerosis might be susceptible to small intestinal mucosal injury from LDA use.

We conducted this multicenter cross-sectional study to clarify the influences of clopidogrel on LDA-induced small intestinal mucosal injuries in actual patients using LDA. We hypothesized that DAPT was a risk factor for worsening LDA-induced small intestinal mucosal injury based on previous reports.^(5,13,15)^ The study population was limited to a single disease state to increase the consistency of the study subjects’ drug treatment regimens.

## Methods

### Subjects

Eligible patients were those aged over 20-years-old at the time of providing consent, had undergone PCI for ischemic heart disease, had continued LDA use, and had freely provided informed consent based on their full understanding of the protocol. The exclusion criteria were as follows: 1) pregnancy; 2) history of intestinal obstruction or suspected gastrointestinal obstruction; 3) history of upper gastrointestinal or small intestinal surgery; 4) use of non-steroidal inflammatory drugs (NSAIDs) within 4 weeks of starting the study; 5) history of small intestinal disease; 6) a lack of consent for the required surgery, if the capsule endoscope was retained within the body; 7) an implanted pacemaker; 8) a change, reduction, or increase in antithrombotic drugs within 2 weeks of study participation; and 9) determination by the investigator (at his/her discretion) that the subject was ineligible for participation in the study for any other reason.

This study was conducted prospectively at Osaka Medical College Hospital and Hokusetsu General Hospital between September 2014 and December 2015. The study was conducted in accordance with the 1975 Declaration of Helsinki (as revised in 1983), and the protocol was approved by the ethics review committee at Osaka Medical College (No. 1530-01) and was registered in the University hospital Medical Information Network Clinical Trial Registry (UMIN000014853).

### Study design

This multicenter cross-sectional study was conducted to compare the adverse effects of DAPT on LDA-induced small intestinal mucosal injury in patients after PCI for coronary stenosis. The subjects were examined for medical history, use of drugs, and recent blood analysis data (within 1 month), and underwent capsule endoscopy (CE) for small bowel screening. The subjects were registered outpatients from each hospital who satisfied the above criteria and provided informed consent.

Patients were grouped into DAPT (including clopidogrel) and non-DAPT patients to compare the small intestinal endoscopic findings as the primary endpoint. As a secondary endpoint to explore other risk factors of LDA-induced small intestinal mucosal injury in patients who experienced PCI, patients were grouped and compared as follows: PPI users vs PPI-free patients. The relationship between the LDA-use period and small intestinal endoscopic findings was also examined. Multivariate analysis was performed on the possible affecting factors of LDA-induced mucosal injury, which were age, sex, DAPT, PPI, gastric mucoprotective drug, and LDA-use period.^(11,15,21)^

### Evaluation of small intestinal lesions based on capsule endoscopy findings

**Fig. 1:**
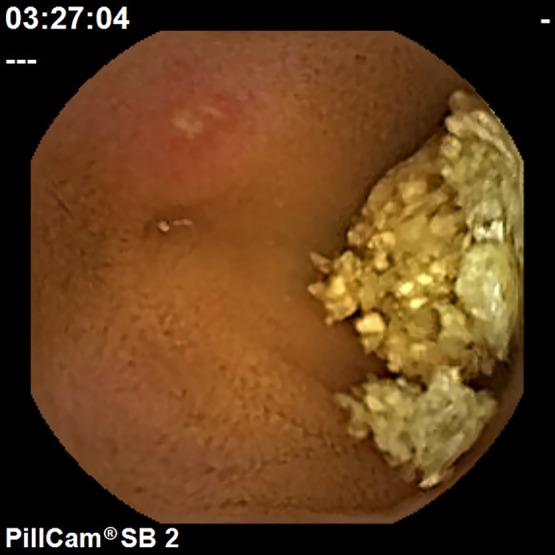
Typical endoscopic findings of small intestinal erosion.

**Fig. 2:**
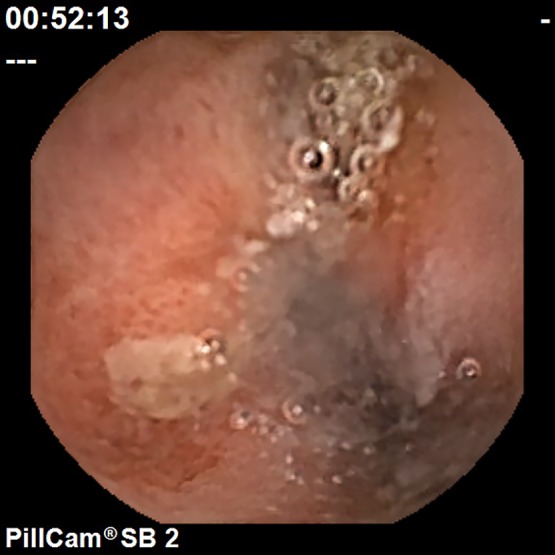
Typical endoscopic findings of small intestinal ulcer.

Small intestinal lesions were examined using a PillCamSB2 (Covidien, Dublin, Ireland), a CE device specifically designed for the small intestine. The CE findings of the small intestinal mucosal injury were evaluated to identify mucosal breaks (erosion and ulcer). In addition, if bleeding or stenosis were detected, they were appended to findings concerning the mucosal injuries. A mucosal break was defined as a defect in the normal villus mucosa, based on the classifications reported by Fujimori *et al.*^(22)^ and Niwa *et al.*^(23)^ with slight modifications. Typical CE findings are shown in Fig. 1 and 2. Evaluation was based on the number of mucosal breaks, and the rate of positive small intestinal mucosal injuries.

### Statistical analysis

The results are presented as number (percentage) or mean ± SD for quantitative data. For continuous and categorical variables, the statistical significance of differences between the groups was determined using the Mann-Whitney *U* test. For binary variables, the statistical significance of differences between the groups was determined using the Pearson’s chi-squared test. All reported *p* values are two-sided, and values <0.05 were considered statistically significant. Analyses were conducted using the JMP^®^ Pro 13 software (SAS Institute Inc., Cary, NC).

## Results

### Patient characteristics

Within the study period, 51 patients who had a history of PCI and were currently using LDA were enrolled. Data from six subjects in whom the entire small intestine could not be visualized via CE were removed from the analysis. Therefore, the analysis was performed using data from 45 patients. Patient characteristics are shown in Table 1. Because blood hemoglobin data were missing in three cases, blood hemoglobin level analysis was done in 42 patients. Of the 45 included patients, 10 patients were using clopidogrel (DAPT group: LDA + clopidogrel, *n* = 8; LDA + clopidogrel + warfarin, *n* = 2), and 35 were not using clopidogrel (non-DAPT group: LDA alone, *n* = 31; LDA + warfarin, *n* = 3; LDA + cilostazol, *n* = 1). Moreover, 23 (51%) patients had a LDA-induced small intestinal mucosal injury, and the mean number of mucosal injuries was 4.0 ± 11.3. There were no significant group differences for patient characteristics between small intestinal injury-positive and negative patients (Table 1).

### Adverse effects of clopidogrel on low-dose aspirin-induced small intestinal mucosal injury in the patients after percutaneous coronary intervention: the primary endpoint

Blood hemoglobin level analysis was performed in 32 subjects in the non-DAPT group, because blood hemoglobin data were missing in three cases. There were no significant differences for sex, age, PPI use, LDA-use period, gastric mucoprotective drug use, blood hemoglobin level, or comorbidities (hypertension and diabetes mellitus) between DAPT patients and non-DAPT patients (Table 2). Moreover, there were no significant group differences for the CE findings, which included the rate of positive small intestinal mucosal injury and the number of mucosal breaks (Table 3).

### Impact of proton pump inhibitors on low-dose aspirin-induced small intestinal mucosal injury: the secondary endpoint

There were no significant differences for sex, age, gastric mucoprotective drug use, blood hemoglobin level, or comorbidities (hypertension and diabetes mellitus) between PPI users and PPI-free patients. The LDA-use period was significantly longer in PPI-free patients than in PPI users (Table 4). Additionally, there were no significant group differences for the CE findings (Table 5).

### Relationship between low-dose aspirin-use period and small intestinal endoscopic findings

**Fig. 3:**
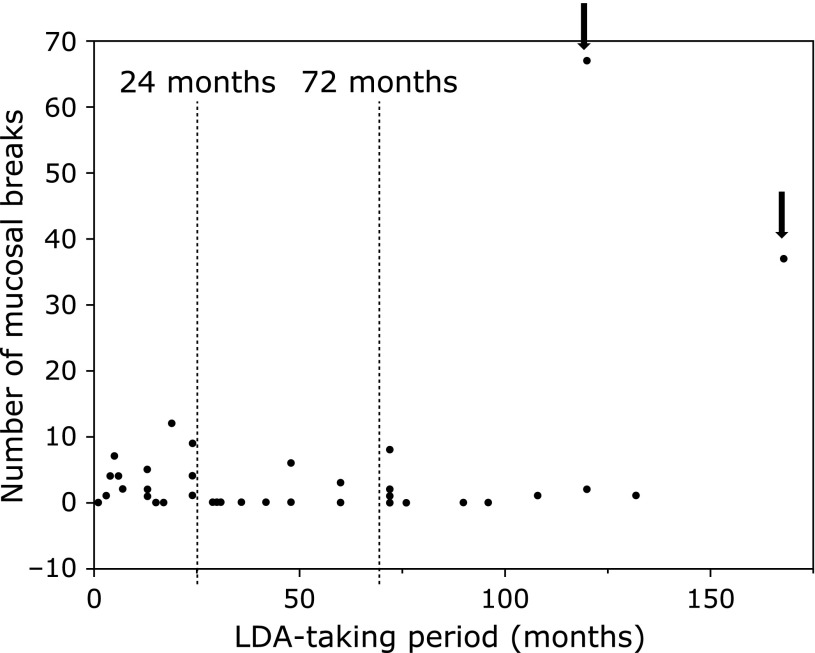
Relationship between LDA-use period and small intestinal endoscopic findings. Patients were divided at the tertiles (24 and 72 months) of the LDA-use period. There were extremely high numbers of LDA-induced small intestinal mucosal breaks in two patients who used LDA for ≥72 months (arrows).

The relationship between LDA-use period and small intestinal endoscopic findings are shown in Table 6 and Fig. 3. Patients were divided at the tertiles (24 and 72 months) of the LDA-use period. For the analysis of LDA-use period based on 24 months, those who used LDA ≤24 months had a significantly higher rate of mucosal breaks and mean number of mucosal breaks than those who used LDA >24 months (Table 6). The analysis of LDA-use period based on 72 months yielded no significant differences. However, of note, there were extremely high numbers of LDA-induced small intestinal mucosal breaks in two patients who used LDA for ≥72 months (Fig. 3).

### Risk factors of low-dose aspirin-induced small intestinal mucosal injury in patients who underwent percutaneous coronary intervention

Multivariate analysis including potential confounding factors was performed to identify risk factors of LDA-induced small intestinal injury (Table 7). The possible risk factors of LDA-induced small intestinal mucosal injury were sex (male), age (>65 years), DAPT use, PPI use, LDA-use period (≤24 months), and the reported suppressor was gastric mucoprotective drugs use. This multivariate analysis revealed that a LDA-use period of ≤24 months was a significant risk factor of LDA-induced small intestinal mucosal injury occurrence.

## Discussion

Although several studies on LDA-induced small intestinal mucosal injury in healthy volunteers have been reported, investigations in actual patients are rare. This study was conducted to compare the adverse effects of DAPT on LDA-induced small intestinal mucosal injury in patients after PCI for coronary stenosis.

The reduction of prostaglandin production in the intestinal mucosa by suppression of cyclooxygenase-1 activity is known to be the main cause of LDA-induced intestinal mucosal injury.^(12)^ Since clopidogrel is not involved in cyclooxygenase-1 activity, the intestinal mucosa is not injured because prostaglandin production is not suppressed. However, a retrospective study by Shiotani *et al.*^(15)^ concluded that DAPT exacerbates small intestinal mucosal injury. Conversely, we speculated that LDA-induced small intestinal mucosal injury may be exacerbated by the degradation of the intestinal environment due to various factors, including changing intestinal flora or severe arteriosclerosis requiring PCI. The short-term LDA users (within 24 months) had significantly more severe LDA-induced small intestinal lesions than long-term LDA users (over 24 months). The secondary prevention for ischemic heart disease, which included not only drug medication but also improvement of patients’ lifestyle (such as eating, exercise, and smoking habits), may be involved in improving small intestinal mucosal injury. The timing of beginning LDA medication overlaps with an unstable state of ischemic heart disease or the initiation of DAPT immediately after PCI. In other words, the risk of small intestinal bleeding may increase in the period of unstable ischemic heart disease. Kawai *et al.*^(24)^ reported that LDA-induced gastrointestinal complications were reduced at 7 days after the start of LDA treatment; this phenomenon was concluded to represent adaptation. Kojima *et al.*^(25)^ reported that NSAID-induced small intestinal mucosal injuries were not aggravated after 2 weeks of treatment, suggesting that mucosal adaptation may occur in the small intestine. In the present study, the finding that small intestinal mucosal injury was milder among long-term LDA users may be related to adaptation effects. However, it was necessary to further investigate the two patients with a LDA-use period ≥72 months who had extremely high numbers of LDA-induced small intestinal mucosal breaks. Although one of the two patients had been using drugs and had improved his lifestyle as directed by his doctor, he had undergone PCI four additional times after the first PCI. Alternatively, the second patient had also followed his doctor’s instructions and additional PCI was not necessary. Both of these patients had controlled hypertension and diabetes. Therefore, according to the findings of these patients, there might be an unknown risk factor of LDA-induced small intestinal mucosal injury. Additionally, it is important to remember that the rate of small intestine mucosal injury occurrence was over 30%, even after 24 months of using LDA, which further suggests that other factors may affect injury incidence.

In a laboratory rat study by Wallace, *et al.*^(26)^ PPIs exacerbated NSAID-induced small intestinal mucosal injury. Additionally, Endo *et al.*^(21)^ reported that combined LDA and PPI use appeared to increase the risk of small intestinal mucosal injury. Watanabe *et al.*^(27)^ reported that PPI use was associated with severity of NSAIDs-induced damage, but not prevalence of such damage. However, the results of the present analysis suggest that PPI does not influence small intestinal mucosa in actual patients. We do not deny that concurrent use of PPI or clopidogrel worsens LDA-induced small intestinal mucosal injury in low-risk subjects, such as healthy volunteers. In this study, we focused on high-risk subjects who were patients who had experienced PCI procedure for coronary stenosis. Half of these patients had small intestinal mucosal injury regardless of PPI use; thus, we consider that administering PPI or clopidogrel in these patients does not significantly affect the incidence of small intestinal endoscopic findings. Accordingly, PPI is considered safe for the treatment of small intestinal mucosa in LDA users after PCI.

The limitations of this study are as follows: (1) it was a single-arm study, and (2) the number of short-term LDA users was small. It is necessary to conduct a prospective, double-arm study comparing short-term and long-term LDA users to clarify whether the period of LDA administration is a risk factor of LDA-induced small intestinal mucosal injury. Although this study was performed in a cohort of patients who experienced PCI procedure for coronary stenosis, LDA-use period of these patients was not consistent across the cohort. Therefore, it was necessary to analyze the relationship between small intestinal lesions and LDA-use period. In the future, clinical trials should focus on usage periods of not only LDA, but also PPI and clopidogrel, which can also be identified as a limitation of the present clinical trial.

In conclusion, DAPT did not affect the incidence of LDA-induced small intestinal mucosal injuries, and PPI did not worsen LDA-induced small intestinal mucosal injury. Patients who used LDA within the last 24 months had significantly more LDA-induced small intestinal mucosal lesions. It is necessary for clinicians to remain cognizant of small intestinal bleeding in patients who underwent PCI within 24 months.

## Figures and Tables

**Table 1:** Patient characteristics and the comparison between small intestinal injury-positive and negative patients

	Total patients (*n* = 45)	Small intestinal mucosal injury (+) (*n* = 23)	Small intestinal mucosal injury (–) (*n* = 22)	*p* value
Sex (male/female)	41/4	22/1	19/3	0.27^b^
Age (years)	69.9 ± 8.1	71.0 ± 9.5	68.7 ± 6.4	0.11^c^
Antiplatelet therapy for post-PCI: DAPT	10, 22.2%	5, 21.7%	5, 22.7%	0.94^b^
Prevention for gastric mucosal injury: PPI	22, 48.9%	11, 47.8%	11, 50.0%	0.88^b^
Prevention for gastric mucosal injury: gastric mucoprotective drug	3, 6.7%	2, 8.7%	1, 4.6%	0.58^b^
LDA-use period (months)	52.3 ± 41.0	54.7 ± 51.7	49.7 ± 26.5	0.59^c^
Blood hemoglobin (g/dl)^a^	14.3 ± 1.54	14.3 ± 1.1	14.2 ± 1.9	0.75^c^
Comorbidity: Hypertension	30, 66.7%	16, 69.6%	14, 63.4%	0.67^b^
Comorbidity: Diabetes mellitus	11, 24.4%	4, 17.4%	7, 31.8%	0.26^b^

Data are presented as number; number, percentage; or mean ± SD. +, positive; –, negative; PCI, percutaneous coronary intervention; DAPT, dual antiplatelet therapy; PPI, proton pump inhibitor; LDA, low-dose aspirin. ^a^Blood hemoglobin level analysis was done in 42 subjects (+, *n* = 22; –, *n* = 20), because blood hemoglobin data were missing in three cases. ^b^Pearson’s chi-squared test. ^c^Mann-Whitney *U* test.

**Table 2:** Patient characteristics based on dual antiplatelet therapy use

	DAPT (*n* = 10)	Non-DAPT (*n* = 35)	*p* value
Sex (male/female)	8/2	33/2	0.16^b^
Age (years)	72.0 ± 5.8	69.3 ± 8.7	0.42^c^
Prevention for gastric mucosal injury: PPI	5, 50%	17, 48.6%	>0.94^b^
Prevention for gastric mucosal injury: gastric mucoprotective drug	1, 10%	2, 5.71%	0.73^b^
LDA-use period (months)	34.3 ± 35.4	57.4 ± 41.4	0.06^c^
Blood hemoglobin (g/dl)	13.8 ± 1.9	14.4 ± 1.4^a^	0.73^c^
Comorbidity: Hypertension	7, 70%	23, 65.7%	0.80^b^
Comorbidity: Diabetes mellitus	2, 20%	9, 25.7%	0.71^b^

Data are presented as number; number, percentage; or mean ± SD. DAPT, dual antiplatelet therapy; PPI, proton pump inhibitor; LDA, low-dose aspirin. ^a^Blood hemoglobin level analysis was done in 32 subjects, because blood hemoglobin data were missing in three cases. ^b^Pearson’s chi-squared test. ^c^Mann-Whitney *U* test.

**Table 3:** Effects of clopidogrel on low-dose aspirin-induced small intestinal mucosal injury

	DAPT (*n* = 10)	Non-DAPT (*n* = 35)	*p* value
Number of patients with mucosal breaks	5, 50%	18, 51.1%	0.94^a^
Number of mucosal breaks	1.9 ± 2.5	4.6 ± 12.7	0.99^b^

Data are presented as number, percentage or mean ± SD. DAPT, dual antiplatelet therapy. ^a^Pearson’s chi-squared test. ^b^Mann-Whitney *U* test.

**Table 4:** Patient characteristics based on proton pump inhibitor use

	PPI users (*n* = 22)	PPI-free (*n* = 23)	*p* value
Sex (male/female)	21/1	20/3	0.32^b^
Age (years)	71.1 ± 8.6	68.7 ± 7.7	0.18^c^
Prevention for gastric mucosal injury: gastric mucoprotective drug	3, 13.6%	0, 0%	0.07^b^
LDA-use period (months)	39.5 ± 41.8	64.4 ± 37.0	0.01^c^
Blood hemoglobin (g/dl)^a^	14.4 ± 1.7	14.1 ± 1.4	0.32^c^
Comorbidity: Hypertension	15, 68.2%	15, 65.2%	0.83^b^
Comorbidity: Diabetes mellitus	5, 22.7%	6, 26.1%	0.79^b^

Data are presented as number; number, percentage; or mean ± SD. PPI, proton pump inhibitor; LDA, low-dose aspirin. ^a^Blood hemoglobin level analysis was performed in 21 subjects in the PPI users group and 21 subjects in the PPI-free patients group, because blood hemoglobin data were missing in three cases. ^b^Pearson’s chi-squared test. ^c^Mann-Whitney *U* test.

**Table 5:** Effects of proton pump inhibitors on low-dose aspirin-induced small intestinal mucosal injury

	PPI users (*n* = 22)	PPI-free (*n* = 23)	*p* value
Number of patients with mucosal breaks	11, 50%	12, 52.2%	0.88^a^
Number of mucosal breaks	6.5 ± 15.8	1.7 ± 2.4	0.73^b^

Data are presented as number, percentage or mean ± SD. PPI, proton pump inhibitor. ^a^Pearson’s chi-squared test. ^b^Mann-Whitney *U* test.

**Table 6:** Relationship between low-dose aspirin-use period and small intestinal endoscopic findings

	24 months				72 months		
	≤ (*n* = 15)	> (*n* = 30)	*p* value		< (*n* = 29)	≥ (*n* = 16)	*p* value
Number of patients with mucosal breaks^a^	12, 80%	11, 36.7%	0.006		14, 48.3%	9, 56.3%	0.61
Number of mucosal breaks^b^	3.5 ± 3.6	4.3 ± 13.7	0.01		2.1 ± 3.1	7.5 ± 18.3	0.81

Data are presented as number, percentage or mean ± SD. ^a^Pearson’s chi-squared test. ^b^Mann-Whitney *U* test.

**Table 7:** Multivariate analysis to identify the risk factors for small intestinal mucosal injury in patients who underwent percutaneous coronary intervention

	Odds ratio	95% confidence interval	*p* value
Age (>65 years)	1.55	0.25–9.62	0.63
Sex (male)	12.2	0.67–258.00	0.11
DAPT (+)	0.63	0.11–3.60	0.61
PPI (+)	0.25	0.05–1.30	0.1
Gastric mucoprotective drug (–)	0.88	0.02–31.9	0.94
LDA-use period (≤24 months)	19.5	2.48–154.00	0.005

DAPT, dual antiplatelet therapy; +, positive; PPI, proton pump inhibitor; –, negative; LDA, low-dose aspirin.
